# Nutritional quality and climate impact of Norwegian adults’ diet classified according to the NOVA system

**DOI:** 10.1186/s12937-024-01066-5

**Published:** 2024-12-31

**Authors:** Christine Slaathaug, Mari Mohn Paulsen, Sepideh Jafarzadeh, Monica Hauger Carlsen, Lene Frost Andersen

**Affiliations:** 1 Department of Nutrition, Institute of Basic Medical Sciences, University of Oslo, Oslo, Norway; 2 Department of Food Safety, Norwegian Institute of Public Health, Oslo, Norway; 3 Centre for Sustainable Diets, Norwegian Institute of Public Health, Oslo, Norway; 4 SINTEF Ocean, Trondheim, Norway

**Keywords:** NOVA classification system, Ultra-processed foods, Climate impact, Nutritional quality

## Abstract

Information about how ultra-processed foods (UPF) contribute to the intake of energy and nutrients and environmental impact is important for future food policies and dietary recommendations. This study assessed the contribution of the four NOVA food groups, including UPF, to energy intake, nutritional quality, and climate impact in Norwegian adults' diet. We used dietary data from 348 participants in the Norkost 4 pilot study, collected through two non-consecutive 24-h dietary recalls. Foods, beverages, and dietary supplements were classified using the NOVA system. The NOVA system has four groups; NOVA 1: *Unprocessed or minimally processed foods*, NOVA 2: *Processed culinary ingredients*, NOVA 3: *Processed foods*, and NOVA 4: UPF. UPF contributed to 48% of the total energy intake, NOVA 3 with 19%, and NOVA 1 with 28%. Within UPF, bread contributed the most to energy intake. Foods in NOVA 1 had the highest nutrient density (expressed as amount of nutrients provided per unit of energy) for protein, fiber, and several essential micronutrients. UPF had the highest nutrient density for added sugar, fats, and sodium. UPF contributed to 32% of total GWP from the diet, while NOVA 1 contributed to 38%. In conclusion, UPF contributed to about half of the energy intake of Norwegian adults, and had lower nutritional quality compared to NOVA 1. UPF also accounted for about one-third of the GWP from the total diet. These findings emphasize the importance of addressing the intake of UPF in dietary policies and recommendations to improve nutritional quality and reduce environmental impact.

**Supplementary Information:**

The online version contains supplementary material available at 10.1186/s12937-024-01066-5.

## Introduction

Traditionally in nutrition research, when investigating the relationship between diet and health outcomes, the focus has been on dietary patterns, foods, energy, or nutrient intake. In recent years, it has been speculated whether the intake of foods with varying degrees of processing may play an independent role in disease development [1]. Several food classification systems based on the degree of food processing have been developed [2, 3]. The NOVA classification system is one of the most well-known systems and introduced the term ‘ultra-processed foods’ [4–6]. The system focuses on the degree and purpose of the food processing and the use of additives and ingredients when classifying food items into the following four different groups; unprocessed and minimally processed foods (NOVA Group 1), processed culinary ingredients (NOVA Group 2), processed foods (NOVA Group 3), and ultra-processed foods (NOVA Group 4), henceforth termed UPF [4, 7, 8].

High intakes of UPF like salty starchy snacks and sugar rich beverages have been associated with lower nutritional quality of the diet and higher intake of unfavorable nutrients such as salt, saturated fat, and added sugars in US, Canada, and Europe [4, 6, 9]. In Norway, consumption of foods according to processing degree has been investigated based on food sales [10] and among pregnant women based on dietary information from a food frequency questionnaire (FFQ) [11]. However, data from the general Norwegian population based on individual dietary information allowing for better food processing classification is lacking.

Processing of food may play an important role in developing an environmentally sustainable food system. On the positive side, food processing enables utilization of by-products and ingredients that would otherwise be wasted [12–14]. Moreover, processed foods generally have longer storage life, which may help to reduce food waste and overcome seasonal availability gaps [15, 16]. In the value chain of a food product, the primary production stage is the largest contributing part to climate impact [17]. However, producing highly processed food, including many plant-based meat and dairy alternatives, often requires more energy and resources than minimally processed plant foods [17]. While plant-based meat and dairy alternatives are more environmentally sustainable than animal-based products, choosing minimally processed options like vegetables and legumes is generally even more sustainable. Moreover, UPF often consists of cheap ingredients from monoculture crops and intensive agricultural systems that lead to high use of pesticides and fertilizers and contribute to biodiversity loss [18]. A review by Anastasiou et al. [19] showed that UPF has a large environmental impact, both on climate, water and land use, waste and biodiversity. Importantly, many minimally processed animal products, like red meat (NOVA 1), also have high environmental impact. This highlights that environmental burden depends not just on processing levels but also on the type and quantity of foods consumed. Additionally, comparing studies is challenging due to varying definitions of UPF. In the present study, we have used UPF defined by the NOVA classification to investigate the climate impact of UPF which has only been explored in a few earlier studies [20–24].

Understanding how UPF contributes to the intake of energy and nutrients, as well as its environmental impact is clearly needed to inform future food policies and dietary recommendations. As mentioned previously there are limited data on the individual intake of UPF in Norway. The aim of the present study was therefore to describe how foods classified according to the NOVA classification system contribute to energy intake, nutritional quality, and climate impact in the diet of Norwegian adults.

## Methods

### Population and dietary data

We based the present study on data from the Norkost 4 pilot study, which included a sample of 348 individuals aged 18–80 living in Norway. The study was conducted from January to May 2021 as a pilot for the larger Norkost 4 study, which was conducted from 2022 to 2023. The National Population Registry drew a nationally representative sample of 800 persons, according to gender, age distribution, educational level, region, and country of birth. Three percent of the persons were excluded due to missing phone numbers, having died, living outside Norway, or residing in institutions. Of the eligible 779 persons, 45% completed two 24-h recalls.

Dietary intake was assessed using two non-consecutive telephone-administered 24-h dietary recalls. These interviews covered both weekdays and weekend days. Participants received a picture booklet of portion sizes along with the invitation, as a guide to report amounts eaten more correctly. We registered dietary intake using an in-house food composition and nutrient calculation system, KBS, developed at the Department of Nutrition, University of Oslo (KBS v.5.4, database version AE-22) [25]. Participants self-reported their educational level, height, and weight. The Body Mass Index (BMI) was estimated based on the self-reported height and weight.

### Food classification

Foods and beverages consumed in the Norkost 4 pilot were systematically classified into the four defined NOVA groups according to the NOVA instructions, based on the list of ingredients and the degree or type of processing [8]. NOVA Group 1 is *Unprocessed or minimally processed foods*, NOVA Group 2 is *Processed culinary ingredients*, NOVA Group 3 is *Processed foods*, and NOVA Group 4 is *Ultra-processed foods* (UPF). Detailed description of the definition can be found elsewhere [4, 7, 8].

As there are gaps in the instructions of the NOVA classification system, meaning that it does not describe in depth all ingredients and processing techniques needed for complete classification of all foods, we made additional specifications about ingredients and additives based on the NOVA group definitions and literature and regulations concerning food additives [26–28]. Full disclosure of additives, ingredients and preparation methods included in each NOVA group used in the present paper, can be found in Supplementary Table S1.

Participants reported consuming 1836 specific food items. We used product information from the manufacturers’ web pages and from Norwegian online food delivery services (Oda (oda.no), Meny (meny.no) and Coop (coop.no)) to find ingredient lists for the foods and beverages consumed, if and when this information was not already available in the food database. From the product ingredient lists, we then evaluated and assigned all 1836 food items into their correct NOVA group. During the 24-h recall interviews, the level of detail of survey foods varied among the participants. Some participants provided brand names for the foods they ate, while others did not, often because the participant did not know the exact brand. If brand name was given, all information about ingredients and processing available in the food composition database or in online resources was used to define the product in one of the four NOVA groups. If brand names were not provided, the food items were categorized into NOVA 1, 2, 3, or 4 based on the food information of the most commonly consumed type of that food item, using data from Norwegian national food surveys. If participants did not know if their food was homemade or industrially produced, we classified these foods as manufactured products. For example, if a participant did not know whether the chocolate cake eaten was homemade or store bought, we classified it as a store-bought manufactured cake. For foods that were available in Norwegian grocery stores both as NOVA 3 products and as UPF products, and brand names were not given, we classified the food intake according to the NOVA group most of the similar food products (> 50%) belonged to. For example, different brands of olives in brine contain different additives, thus some olives in brine were classified as NOVA 3 and others as UPF. If a participant recorded intake of olives in brine without specifying the brand, we categorized the intake as UPF, since the majority (> 50%) of olives in brine products on the Norwegian market contain an additive that qualifies them as UPF product. We classified homemade composite dishes including both UPF ingredients and non-UPF ingredients according to the NOVA group most of the ingredients (> 50%) belonged to. To further quantify different types of UPF products from each other, we assigned each food item to ordinary food categories, such as meat product, vegetables product and so on, within the NOVA group they belonged to [25]. Table S2 presents the subgroup categories within the NOVA groups.

Based on the dietary data in the present study, product declarations, and online product information, we estimated that approximately two-thirds of the breads reported as consumed were UPF, while the remaining one-third were NOVA 3. In the Norkost 4 pilot study, some bread intakes were recorded with generic bread codes, because the participants did not provide enough detailed information for specific classification. These codes only indicated whether the bread was purchased rather than homemade, without providing any information on brand or ingredient list. Therefore, we did a sensitivity analysis for all registrations of bread intake with a generic code to explore how the NOVA classification of these breads could impact the results. First, we calculated the number of eating occasions for each bread type. Some bread types were eaten only a few times, while others were consumed frequently, Then, we stratified the recorded bread intakes as few (1–9), medium (10–49), and many (50–175) eating occasions. Lastly, we did the sensitivity analysis through randomizing one third of the generic bread codes to NOVA 3, and two thirds of the generic bread codes to UPF across the stratified eating occasions.

### Dietary supplements

Vitamin and mineral supplements, including cod liver oil and other omega-3 fatty acid supplements, were excluded from the analyses. We included dietary supplements such as protein bars and protein powder and classified them according to processing degree and ingredient list.

### Climate impact

We used global warming potential (GWP) in kg CO_2_-equivalents for a 100-year time scale, and per 100 g edible food item, as the climate indicator. The climate values were incorporated on food item level in the food database of the KBS food and nutrient calculation system. A detailed description of the climate impact database can be found elsewhere [29]. In short, the climate database was developed in a 4-year long research project where LCA data for food items were compiled from an extensive literature search, including scientific LCA articles and reports. The system boundaries were set to include the most important steps from farm to fork, including primary production, processing, packaging, distribution, wholesale, retail, storage, and consumer preparation, as well as waste along these steps, if reported. Data gaps on household waste and transportation from retail to household were not estimated due to scarce data and uncertain estimations. As the published literature on LCA on food is limited we filled data gaps with estimated climate impact values using the SimaPro software, version 9.0.0.49, with processes from the EcoInvent [30] and the Agri-Footprint [31] databases. Also, LCA data was compiled from other climate databases, mostly the National Institute for Public Health and the Environment in the Netherlands (RIVM) [32]. The GWP data incorporated in the climate database estimated values for the impact of transport and industrial processing. UPF undergo processing of which there were scarce LCA data at the time of compilating the climate impact database. To include estimates of the impact of processing in the estimates, a sensitivity analysis was conducted for the GWP analyses, adding 5% and 10% to the GWP values of UPF products, as rough estimates of the potential missing impact of the processing steps of the food products.

### Analysis and statistics

We present descriptive statistics on food consumption by NOVA groups as a percentage of total energy intake. We express the nutrient density for each NOVA group as energy percentages (E%) for macronutrients and per megajoule (MJ) for micronutrients. We investigated differences in nutrient density across the NOVA 1 and UPF, and within the sensitivity analyses using paired samples T-test for normally distributed variables and Wilcoxon Signed Rank for skewed variables. We evaluated normality for all variables using visual interpretation of histograms, including boxplots, and median versus mean. Due to skewed distributions for several variables, we reported all variables within tables as median, 25th percentile (P25), 75th percentile (P75), and mean. Variables are reported as median in the text, except the background characteristics of the participants, which were normally distributed. We performed statistical analyses using IBM SPSS Statistics version 29. The statistical significance level was set to 1% and not 5% due to multiple testing.

### Ethics

The Norwegian Centre for Research Data (NSD) approved The Norkost 4 pilot study, reference number 370209. The Regional Committees for Medical and Health Research Ethics in Norway (REK) determined that it was not necessary to conduct an ethical assessment of the project. We conducted the Norkost 4 pilot study according to the guidelines in the Declaration of Helsinki. All participants gave a verbal informed consent when contacted by the interviewers, and the consent was registered in an in-house log-system. 

## Results

### Characteristics

A total of 348 (45%) of the invited participants completed the study. Characteristics of the participants are presented in Table 1. The mean age and BMI for the study sample were 48 years and 25.9 kg/m^2^, respectively. There was a slightly higher proportion of participants with high education (55%), defined as university, college, or higher, compared to low education (45%), defined as high school, technical school, trade school, or lower. Mean energy intakes were 10.4 MJ for men and 8.0 MJ for women.

**Table 1:** Characteristics of the participants in the present study

Characteristic	All (*n* = 348)	Men (*n* = 171)	Women (*n* = 177)
Age, years, mean (SD)	48 (17)	50 (17)	47 (16)
Educational level			
Low education, %	45	47	43
High education, %	55	53	57
BMI, kg/m^2^, mean (SD)	25.9 (4.4)	26.1 (3.7)	25.7 (4.9)
Energy intake, MJ, mean (SD)	9.2 (3.2)	10.4 (3.3)	8.0 (2.5)

Low education includes high school, technical school, trade school or lower

High education includes university, college, or higher

BMI (Body Mass Index) was missing for 4% of the women, 2% of the total sample

### Contributions from the NOVA groups to energy intake

The contribution of energy from foods classified according to the four NOVA groups is presented in Table 2. NOVA 1, NOVA 3, and UPF contributed to 28%, 19%, and 48% of the total energy intake, respectively. Of the food groups in NOVA 1, *Fruit, vegetables, and potatoes* contributed to 9.7% of total energy intake, followed by *Fish, eggs, and meat*, which contributed 5.8%. Among the food groups in NOVA 3, *Dairy products* were the most important contributor to total energy intake, with a median contribution of 5.6%. For food groups in UPF, *Bread* was the most important contributor to total energy intake with a median contribution of 9.5%, followed by *Meat and meat products*, which contributed 6.6%. Supplementary Table S3 shows the participants absolute intake in grams across food groups according to the four NOVA groups.

**Table 2:** The contribution of energy from foods classified according to the four NOVA groups among Norwegian adults (*n* = 348)

Food group	Percentage of total energy intake			
	Median (P25, P75) mean			
	NOVA 1	NOVA 2	NOVA 3	UPF
**Total intake**	28 (19, 38) 30	1.8 (0.0, 4.8) 3.4	19 (10, 28) 20	48 (33, 60) 47
**Fruits, vegetables**,** and potatoes**	9.7 (5.1, 15) 11	-	0.0 (0.0, 2.2) 1.6	1.0 (0.0, 3.3) 2.4
Fruits, berries, nuts, and seeds	4.8 (1.7, 9.2) 6.1	-	0.0 (0.0, 0.9) 1.1	0.0 (0.0, 1.3) 0.8
Vegetables	1.6 (0.6, 3.5) 2.8	-	0.0 (0.0, 0.0) 0.4	0.0 (0.0, 0.2) 0.5
Legumes	0.0 (0.0, 0.0) 0.2	-	0.0 (0.0, 0.0) 0.2	0.0 (0.0, 0.0) 0.2
Potatoes	0.0 (0.0, 3.6) 2.3	-	-	0.0 (0.0, 0.0) 0.8
**Bread, cake, and other grain products**	2.9 (0.0, 8.1) 5.5	0.0 (0.0, 0.0) 0.0	3.9 (0.0, 9.4) 6.2	15 (8.7, 24) 17
Bread	-	-	1.6 (0.0, 7.0) 4.6	9.5 (3.4, 15) 10
Bread, < 25% whole grains	-	-	0.0 (0.0, 0.0) 0.8	0.0 (0.0, 1.5) 1.7
Bread, ≥ 25% whole grains	-	-	0.0 (0.0, 5.6) 3.8	7.5 (1.4, 14) 8.7
Flour, cereals, noodles, and other grain products	3.0 (0.0, 8.1) 5.5	0.0 (0.0, 0.0) 0.0	0.0 (0.0, 0.0) 0.8	0.0 (0.0, 4.0) 2.5
Cakes and pastry	-	-	0.0 (0.0, 0.0) 0.8	0.0 (0.0, 6.2) 4.0
**Fish, eggs, and meat**	5.8 (1.4, 12) 8.2	-	0.0 (0.0, 3.1) 2.6	9.5 (2.8, 18) 12
Fish and fish products	0.0 (0.0, 0.0) 1.8	-	0.0 (0.0, 0.0) 1.2	0.0 (0.0, 2.1) 2.0
Eggs	0.0 (0.0, 3.8) 2.4	-	-	-
Meat and meat products	0.0 (0.0, 6.0) 4.0	-	0.0 (0.0, 1.1) 1.4	6.6 (1.0, 16) 10
**Dairy products**	2.1 (0.0, 5.7) 4.0	-	5.6 (2.2, 10) 7.0	0.6 (0.0, 3.8) 2.4
Milk	0.0 (0.0, 2.3) 1.7	-	0.0 (0.0, 0.5) 1.1	0.0 (0.0, 0.0) 0.1
Yoghurt and fermented milk	0.0 (0.0, 0.5) 0.7	-	0.0 (0.0, 0.0) 0.0	0.0 (0.0, 0.0) 0.9
Cheese	-	-	4.3 (1.5, 8.4) 5.9	0.0 (0.0, 0.0) 0.5
Other dairy products	0.0 (0.0, 0.9) 1.6	-	-	0.0 (0.0, 0.0) 0.8
**Beverages**	0.1 (0.0, 0.2) 0.5	-	0.0 (0.0, 3.8) 2.5	0.1 (0.0, 2.6) 2.2
Non-alcoholic beverages	0.1 (0.0, 0.2) 0.5	-	0.0 (0.0, 0.0) 0.5	0.1 (0.0, 2.4) 2.0
Alcoholic beverages	-	-	0.0 (0.0, 2.5) 2.0	0.0 (0.0, 0.0) 0.2
**Vegetable oils and fats**	-	1.1 (0.0, 4.0) 2.8	0.0 (0.0, 0.0) 0.3	2.2 (0.0, 4.8) 3.2
Vegetable oils	-	0.0 (0.0, 0.5) 0.8	-	-
Butter and margarine	-	0.0 (0.0, 3.4) 1.9	-	0.0 (0.0, 2.8) 1.8
Other fats	-	0.0 (0.0, 0.0) 0.0	0.0 (0.0, 0.0) 0.3	0.0 (0.0, 2.1) 1.4
**Miscellaneous items**	0.0 (0.0, 0.0) 0.1	0.0 (0.0, 0.6) 0.6	-	4.8 (0.8, 10) 7.2
Sugars and sweets	-	0.0 (0.0, 0.6) 0.6	-	2.2 (0.0, 6.2) 4.2
Salty snacks	-	-	-	0.0 (0.0, 1.4) 2.0
Other	0.0 (0.0, 0.0) 0.1	0.0 (0.0, 0.0) 0.0	-	0.0 (0.0, 0.7) 1.0

Full disclosure of food items included in each food group can be found in Supplementary Table S1

*Abbreviations*: *NOVA 1* NOVA Group 1, *NOVA 2* NOVA Group 2, *NOVA 3* NOVA Group 3, *P25* 25th percentile, *P75* 75th percentile, *UPF* Ultra-processed foods (NOVA Group 4)

Results from the sensitivity analysis for bread are presented in Table 3. A decrease in the percentage of total energy intake from UPF (48% vs. 43%) (*p* < 0.01) and an increase in the percentage of total energy intake from NOVA 3 (19% vs. 24%) (*p* < 0.01) were found in the sensitivity analysis compared to the main analysis. There was a decrease in energy from bread classified as UPF (9.5% vs 4.4%) (*p* < 0.01) in the sensitivity analysis compared to the main analysis, the largest decrease was observed for *Bread,* ≥ *25% wholegrain*. The opposite was observed for bread classified as NOVA 3 with an increase (1.6% vs. 7%) (*p *< 0.01).

**Table 3:** Percentage of total energy intake from all foods and from bread based on main analysis and sensitivity analysis (*n* = 348)

Food group	Percentage of total energy intake			
	Median (P25, P75) mean			
	Main analysis		Sensitivity analysis^a^	
	NOVA 3	UPF	NOVA 3	UPF
**Total intake**	19 (10, 28) 20	48 (33, 60) 47	24 (14, 32) 24**	43 (29, 56) 42**
**Bread, total**	1.6 (0.0, 7.0) 4.6	9.5 (3.4, 15) 10	7.0 (1.6, 13) 8.7**	4.4 (0.0, 11) 6.3**
Bread, < 25% wholegrains	0.0 (0.0, 0.0) 0.8	0.0 (0.0, 1.5) 1.7	0.0 (0.0, 0.0) 1.1**	0.0 (0.0, 0.0) 1.4**
Bread, ≥ 25% wholegrains	0.0 (0.0, 5.6) 3.8	7.5 (1.4, 14) 8.7	5.9 (0.0, 12) 7.5**	1.9 (0.0, 8.2) 4.9**

Sensitivity analyses were conducted using Paired Samples T-Test or Wilcoxon Signed-Rank Test depending on the normality or skewness of the data

In the statistical analysis NOVA 3 main analysis were compared with NOVA 3 sensitivity analysis, and UPF main analysis with UPF sensitivity analysis

*Abbreviations:* *NOVA 3* NOVA Group 3, *UPF* Ultra-Processed Foods (NOVA Group 4), *P25* 25th percentile, *P75* 75th percentile

** Statistical significance at *p* < 0.01

^a^In the sensitivity analysis, the bread with a generic coding was randomly distributed to one third as NOVA group 3, and two thirds as NOVA group 4, as this represents the approximate distribution of bread in Norwegian online grocery stores

### Nutritional quality

The nutrient density of the total diet and from the four NOVA groups for both main analysis and sensitivity analysis is presented in Table 4 and in Supplementary Table S4. Nutrient density for protein, fiber, vitamin A, folate, vitamin B12, vitamin C, vitamin D, iron, calcium, iodine, and selenium was lower for UPF compared to NOVA 1 (*p* < 0.01). Nutrient density for added sugar, total fat, SFA, MUFA, PUFA, and sodium were higher for UPF compared to NOVA 1 (*p* < 0.01).

**Table 4:** Nutrient density of the total diet and within the NOVA groups among Norwegian adults in main analysis and in sensitivity analysis (*n* = 348)

Nutrient	Nutrient density of total dietary intake Median (P25, P75) Mean	Nutrient density within the NOVA groups Median (P25, P75) Mean						
		Main analysis				Sensitivity analysis		
		NOVA 1	NOVA 2	NOVA 3	UPF	NOVA 3	UPF	
**Protein, E%**	18 (15, 21) 18	22 (16, 29) 23	0.0 (0.0, 0.3) 0.1	20 (15, 27) 22	14 (11, 17) 15**	19 (15, 24) 20 **	14 (11, 18) 15	
**Carbohydrate, E%**	41 (37, 46) 41	44 (34, 54) 44	0.0 (0.0, 16) 17	28 (15, 43) 29	47 (40, 54) 46	37 (23, 49) 36**	44 (36, 51) 43**	
**Added sugar, E%**	5.6 (3.2, 9.1) 6.8	0.0 (0.0, 0.2) 0.4	0.0 (0.0, 0.0) 11	0.1 (0.0, 2.3) 3.3	10 (5.7, 17) 12**	0.2 (0.0, 1.8) 2.6**	12 (5.9, 19) 13**	
**Fiber, g/MJ**	2.8 (2.2, 3.2) 2.9	3.5 (2.4, 5.4) 4.1	0.0 (0.0, 0.0) 0.0	1.8 (0.6, 3.2) 2.2	2.5 (1.8, 3.1) 2.6**	2.6 (1.4, 3.8) 2.7**	2.1 (1.5, 2.9) 2.3**	
**Total fat, E%**	38 (32, 42) 37	29 (20, 38) 30	72 (0, 102) 54	38 (27, 53) 39	37 (30, 44) 38**	32 (23, 43) 34**	41 (31, 49) 41**	
**SFA, E%**	13 (11, 16) 14	8.7 (6.0, 13) 9.8	10 (0.0, 47) 21	17 (9.9, 25) 18	12 (9.0, 15) 13**	14 (8.3, 19) 15**	13 (10, 17) 14**	
**MUFA**^**d**^**, E%**	14 (12, 16) 14	11 (6.2, 15) 11	22 (0, 36) 23	11 (7.8, 16) 13	15 (12, 19) 16**	9.7 (6.7, 14) 11**	16 (12, 21) 17**	
**PUFA**^**e**^**, E%**	6.0 (4.8, 7.2) 6.2	5.2 (3.5, 7.3) 5.5	2.3 (0.0, 9.9) 6.7	3.5 (1.8, 6.0) 4.8	6.4 (4.9, 8.4) 6.9**	3.5 (2.2, 5.7) 4.8	6.8 (5.0, 9.2) 7.3**	
**Vitamin A, µg/MJ**	74 (51, 108) 92	102 (61, 165) 142	0.0 (0.0, 218) 90	43 (24, 74) 54	46 (20, 95) 76**	36 (18, 57) 43**	50 (22, 112) 87**	
**Folate, µg/MJ**	29 (23, 36) 30	48 (34, 66) 55	0.0 (0.0, 0.0) 0.1	25 (19, 31) 26	19 (15, 26) 23**	25 (20, 30) 25	19 (14, 26) 22**	
**Vitamin B12, µg/MJ**	0.7 (0.5, 0.9) 0.8	0.7 (0.4, 1.1) 1.0	0.0 (0.0, 0.0) 0.0	0.9 (0.5, 1.4) 1.1	0.4 (0.3, 0.7) 0.6**	0.7 (0.4, 1.2) 0.9**	0.5 (0.3, 0.7) 0.7**	
**Vitamin C, mg/MJ**	10 (6.5, 17) 12	29 (16, 53) 38	0.0 (0.0, 0.0) 0.0	0.2 (0.0, 1.8) 4.8	3.2 (1.4, 6.0) 4.3**	0.3 (0.0, 1.6) 2.2**	3.5 (1.6, 6.5) 4.7**	
**Vitamin D, µg/MJ**	0.5 (0.3, 0.8) 0.7	0.5 (0.1, 1.2) 0.9	0.0 (0.0, 2.8) 1.1	0.2 (0.0, 0.9) 0.7	0.3 (0.1, 0.6) 0.5**	0.2 (0.0, 0.8) 0.6**	0.3 (0.1, 0.7) 0.5**	
**Iron, mg/MJ**	1.1 (1.0, 1.3) 1.2	1.5 (1.1, 1.9) 1.6	0.0 (0.0, 0.0) 0.0	0.9 (0.5, 1.4) 1.0	1.0 (0.8, 1.3) 1.1**	1.1 (0.7, 1.4) 1.1**	1.0 (0.7, 1.3) 1.0**	
**Calcium, mg/MJ**	107 (82, 135) 111	109 (70, 169) 139	0.0 (0.0, 8.2) 5.8	192 (106, 316) 223	59 (38, 91) 70**	158 (92, 256) 186**	62 (39, 94) 74**	
**Iodine, µg/MJ**	14 (9.7, 24) 20	17 (10, 30) 30	0.2 (0.0, 5.2) 2.8	15 (8.8, 30) 25	7.0 (3.9, 16) 15**	12 (7.2, 23) 21**	7.6 (4.3, 17) 16**	
**Selenium, µg/MJ**	5.1 (4.0, 7.3) 6.3	7.3 (3.9, 12) 8.7	0.0 (0.0, 0.0) 0.1	5.2 (3.6, 7.8) 7.6	3.5 (2.5, 5.0) 4.8**	4.5 (3.2, 7.0) 6.8**	3.6 (2.5, 5.3) 5.0**	
**Sodium (Na), mg/MJ**	330 (265, 409) 347	137 (89, 237) 194	97 (0.0, 203) 454	325 (245, 507) 480	395 (294, 478) 406**	368 (274, 499) 437	382 (285, 480) 410**	

Nutrient density is described as percentage of energy intake, or as gram/mg/µg pr Mega Joule (MJ)

In the sensitivity analysis, one third of the bread with a generic coding, was randomly distributed to one third as NOVA group 3, and two thirds as NOVA group 4, as this represent the approximate distribution of bread in Norwegian online grocery stores

Statistical analyses were conducted using Paired Samples T-Test or Wilcoxon Signed-Rank Test depending on the normality or skewness of the data. In the statistical analysis NOVA 1 main analysis were compared with UPF main analysis, NOVA 3 main analysis with NOVA 3 sensitivity analysis, and UPF main analysis with UPF sensitivity analysis

*Abbreviations*: *NOVA 1* NOVA Group 1, *NOVA 2* NOVA Group 2, *NOVA 3* NOVA Group 3, *UPF* Ultra-Processed Foods (NOVA Group 4), *P25* 25th percentile, *P75* 75th percentile

*Abbreviations*: *SFA* Saturated fatty acids, *MFA* Monounsaturated fatty acids, *PFA* Polyunsaturated fatty acids

^**^ Statistically significant difference at *p* < 0.01

For the sensitivity analysis for bread shown in Table 4, nutrient density in NOVA 3 decreased for protein, added sugar, total fat, SFA, MUFA, vitamin A, B12, C, D, calcium, iodine, and selenium, compared to NOVA 3 in the main analysis. Conversely, nutrient density for carbohydrate, fiber, iron, and sodium increased (*p* < 0.01). For UPF, the nutrient density increased in the sensitivity analysis for the nutrients where NOVA 3 showed a decrease, and vice versa. The only exceptions were protein, which remained unchanged, and folate, which decreased compared to the main analysis of UPF (*p* < 0.01).

### Climate impact

On average, NOVA 1 contributed with 1.5 kg CO_2_-equivalents, UPF with 1.2 kg CO_2_-equivalents and NOVA 3 with 0.8 kg CO_2_-equivalents per person per day. In the sensitivity analysis of 5% and 10%, UPF contributed with 1.3 and 1.4 kg CO_2_-equivalents per person per day, respectively. Total GWP of the diet was 4.1 kg CO_2_-equivalents in the main analysis, and 4.2 kg CO_2_-equivalents and 4.3 kg CO_2_-equivalents per person per day in the 5% and 10% sensitivity analysis, respectively.

**Fig. 1:**
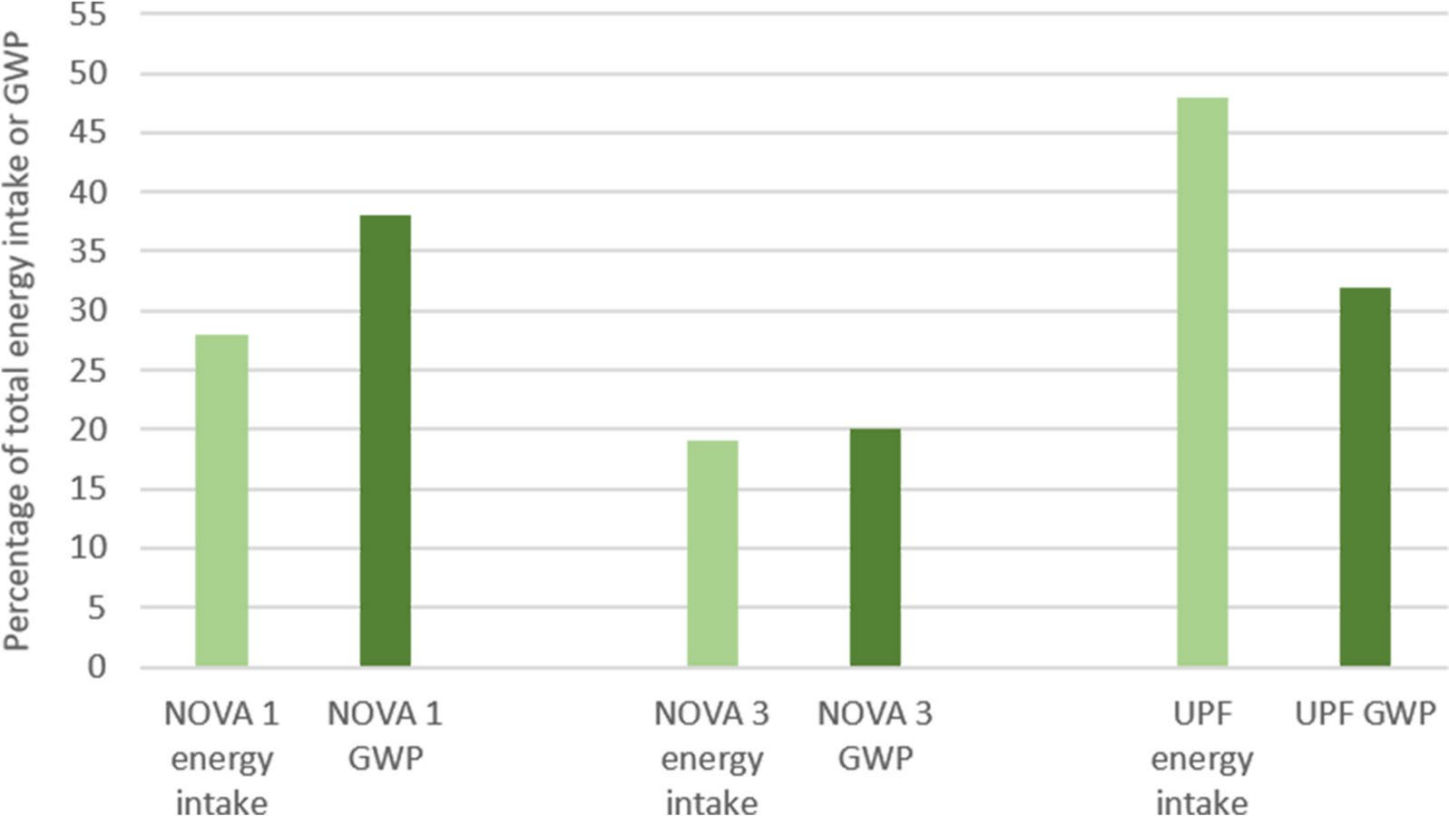
Contribution from the NOVA 1 and UPF to total energy intake and total global warming potential (GWP) in main analysis. Measured as percentage of the total intake and impact (*n* = 348). Abbreviations: NOVA 1; NOVA group 1, UPF; Ultra-processed foods (NOVA group 4), GWP; Global warming potential

Figure 1 shows that NOVA 1 contributed to 28% of the energy intake and 38% of the GWP in the diet. UPF contributed to almost half of the energy intake (48%) in the diet, while the contribution to GWP was about one third (32%) in the main analysis. The 5% and 10% sensitivity analysis for UPF resulted in UPF contributing to 34% and 35% of total GWP, respectively (not shown in the figure). The percentage for NOVA 1 changed from 38 to 37% in both sensitivity analyses, while the percentage for NOVA 3 remained unchanged.

## Discussion

In the present study, we assessed the intake of foods according to the NOVA groups among Norwegian adults, and the contribution of each NOVA group to nutritional quality and climate impact. UPF contributed to almost half of the energy intake, while foods from NOVA 1 contributed to one third of the energy. When we re-classified bread from UPF to NOVA 3, the energy contribution from UPF was changed with 5% percentage points (from 48 to 43%). The NOVA 1 foods consumed had a significantly higher nutrient density for protein, fiber, and all micronutrients, except for sodium, compared to foods consumed within the UPF category. Conversely, the nutrient density of added sugar, total fat, SFA, MUFA, PUFA and sodium, was significantly lower in NOVA 1 foods compared to UPF. For climate impact, NOVA 1 contributed with a higher proportion of the impact from GWP in both the main analysis and the sensitivity analysis, compared to the other NOVA groups.

UPF contributed to 48% of the total energy intake, which is slightly lower than found in adult populations in the US and UK, where UPF was estimated to contribute to more than 50% of the total energy intake [33]. Two other Norwegian studies based on sales data [10] and on pregnant women [11] found similar results as the present study. NOVA 3 contributed to 19% of the total energy intake in the present study, which is in line with the 22% reported in the study of Norwegian pregnant women [11].

Of the food groups in the present study, bread contributed the most to total energy intake from UPF, followed by meat and meat products. These results contrast with findings from the rest of Europe, where fine bakery wares and soft drinks were found to be the main contributors to energy intake from UPF [34]. It is worth noting that all UPF, including energy-poor items such as sugar-free soft drinks, were included in our analyses. Although the consumption of sugar-free soft drinks is relatively high in Norway, these products contribute little to total energy intake, and their impact on the overall energy contribution from UPF is therefore minimal. Bread is a traditional staple food consumed in several meals in Norway, and especially whole grain bread has strong traditions [35, 36]. This is reflected in our results, as bread with more than 25% wholegrains contributed more to total energy intake from both NOVA 3 and UPF, compared to bread with less than 25% wholegrains.

Foods in the UPF group had higher nutrient densities for added sugar, total fat, and sodium compared to foods in NOVA 1. Foods in the NOVA 1 group had higher nutrient densities for protein, fiber, and all micronutrients except sodium. These findings are consistent with previous findings from other countries, including UK, Belgium, Australia, Mexico, Brazil, US, and Canada [4, 6, 9]. In the sensitivity analysis (Table 4), where more bread intakes were reclassified as NOVA 3, the nutrient density of the NOVA group 3 increased for fiber and iron, and decreased for sugar, fat, and sodium, giving NOVA 3 as a group an improved nutrient profile. The nutrient density for vitamin A, C, and D, as well as calcium, iodine, and selenium decreased as well, which can be explained by bread generally being low in these nutrients. UPF obtained a reduced nutrient profile in the sensitivity analysis, except for the micronutrients that bread is low on, which improved relatively for UPF (vitamin A, C, calcium, iodine and selenium). These results show that the nutrient density associated with a specific NOVA group will vary significantly depending on how the classification of foods are done.

UPF was estimated to contribute to 32% of the total GWP from the diet (median percentage). This was lower than the contribution from NOVA 1 (38%), and higher than from NOVA 3 (20%). UPF also contributed to a relatively higher percentage of energy intake, compared to the percentage of GWP. The opposite was observed for NOVA 1 (Fig. 1). These findings align with a study from the UK, which found that UPF had a lower GHGE per 100 cal compared to NOVA 1 foods [20]. However, this contradicts other research, such as a 2-year longitudinal study involving nearly 6,000 participants from Southern Europe [37]. Our observations could be due to the primary production of produce being the largest contributing part of the food production to climate impact, while post-harvest processing, transport and additional processing have a lower contributing parts of the climate impact of food [38]. This makes the type of ingredients in UPF more important than the level of processing itself. However, GWP is just one indicator of environmental impact. To fully understand how UPF contributes to the environmental footprint, other measures, such as water footprint, land use, and eutrophication must be considered. Moreover, as mentioned earlier, UPF often relies on cheap ingredients from monoculture crops, which can negatively affect environment through high pesticide use, and reduced biodiversity [18]. UPF has also been found to possibly facilitate overconsumption of energy, leading to overweight and obesity [8, 39]. This can negatively affect the overall climate impact of the diet, as overconsumption can lead to unnecessarily high food production. Additionally, a higher prevalence of overweight and obesity could increase the pressure on healthcare systems, thereby increase emissions and the use of natural resources [18, 40]. In addition, NOVA 1 foods, which are often less energy-dense than UPF, are recommended to help prevent energy overconsumption. This is important for maintaining good health and avoiding excessive weight gain, which could lead to non-communicable diseases [41, 42]. Therefore, UPF may not necessarily be a sustainable food group despite low GWP per MJ in a Norwegian setting.

A major strength of the present study was the use of data from two 24-h dietary recalls, which provide enough details about the foods to perform the NOVA classification. Many studies on UPF and NOVA classification have been conducted with dietary data derived from methods such as FFQs that do not give the detailed information needed to classify foods correctly. Another strength is the thorough and systematic approach used in the classification process, ensuring an objective classification of food items, with full disclosure of additives, ingredients, and preparation methods for each NOVA group. Moreover, the use of sensitivity analysis for bread strengthens the present study and provides insights into the uncertainties pertaining to bread classification. A potential weakness of the present study is the low sample size and the low participation rate of 45%, which may limit the generalizability of our results. We found that a higher proportion of the participants in the Norkost 4 pilot study had a higher education compared to the general population. Those who chose not to participate may have consumed other foods than what was reported by the participants.

Even though the NOVA classification system today is the most widely used, it has come under criticism in recent years [43]. NOVA has been criticized for being confusing, inconsistent, and controversial [43–45]. Lack of categorical specifications and conceptual inconsistencies may be challenging when applied in research and practice [46–48]. This was evident also in the present study, where a vast variety of products were classified as UPF by the NOVA classification system, sometimes only due to one ingredient, such as olives in brine containing stabilizer, or granola containing fiber extract. It is questionable if these foods are contributing to the negative health effects associated with UPF, as there is lacking evidence whether these ingredients such as fibers and stabilizers are causing the negative health effects [49]. This raises the question of whether the issue lies more in the specific formulation of these products, rather than the processing itself. Several foods classified as NOVA 1, 2, and 3 have been associated with negative health outcomes as well, such as red meat (classified as NOVA 1) and processed meat (classified as NOVA 3, for example cured meat or ground beef), and high intakes of added sugars (classified as NOVA 2) [50]. One study from UK found the most nutritious and environmentally friendly foods to be distributed throughout all four NOVA groups [20]. Therefore, the usefulness of the NOVA classification and the term UPF is questionable, as it does not necessarily add something unique compared to looking at food based on nutrient profile, food category, or the health outcomes they are associated with [49, 51, 52]. The functionality of the NOVA system to inform dietary guidelines has also been questioned [43, 46], calling for a precise and rigorous standard classification system that differentiates within food groups and considers nutritional quality to limit interpretational uncertainty and risk of misclassification [53]. The inherent limitations of the NOVA classification system necessitate making additional classification criteria when applying the system to food databases. This makes room for subjective interpretations of the classification system between studies and studies have shown large variability in how people with education within nutrition classify foods according to the NOVA system [46]. This variability affects the results and limits direct comparisons between studies and is a limitation to all studies on UPF intake.

If participants did not know whether their food was homemade or industrially produced, the foods were classified according to the manufactured product. This may have led to an overestimation of the contribution from UPF to percentage of energy intake in the main analysis. In the sensitivity analysis, registrations which used generic codes for bread were randomly classified according to the share of bread in the store that were NOVA 3 or UPF. The results showed a significant decrease in the contribution of UPF to total energy intake compared to the main analysis (from 48 to 43%). This emphasizes the importance of having enough details about products consumed to classify foods correctly. It also shows that the chosen method for classification of food, where the NOVA classification system is lacking details, will affect the result.

While NOVA was initially designed to categorize foods based on their level of processing, some argue that its application may not provide additional insights when assessing environmental impact [22]. Although interventions aimed at replacing UPFs with NOVA 1 foods can often improve human health, they do not necessarily result in a reduced environmental impact [22]. Our sensitivity analysis illustrated that the association between UPF and environmental impact is vulnerable to the NOVA classification. This should be considered when interpreting the results and could be identified as a weakness in the approach to linking UPF with environmental impact. In the present study, we have only included data on climate impact. The climate data included in the compiled LCA database used in this study come with several well-known limitations: LCA data availability can vary, and there is limited data on the processing stages of the LCA.

The findings in the present study fit within a broader context of ongoing debates about the utility of food classification systems like NOVA in assessing environmental outcomes. They underscore the complexity of linking food processing levels directly with environmental sustainability metrics. Given these complexities, our study adds to the body of evidence suggesting that a more nuanced approach may be necessary. Since our study only included data on climate impact, further research is needed to provide a comprehensive understanding of how UPF affects environmental sustainability. Future studies should expand environmental metrics beyond climate impact to include, for example, water usage, land use, and biodiversity loss. Additionally, improving LCA databases to cover more detailed data on food processing can reduce the uncertainty of the results. Investigating policy interventions may also be crucial for designing strategies that promote both health and environmental sustainability.

## Conclusion

This is the first study in Norway to determine intake, nutritional quality, and climate impact of food according to the NOVA groups using 24-h dietary recalls on a sample of the general adult population. UPF was the NOVA group contributing the most to energy intake, with 48% in the main analysis and 43% in the sensitivity analysis. On group level, NOVA 1 foods were found to have better nutritional quality than UPF, but it is important to keep in mind the large variations of foods within each NOVA group. NOVA 1 foods contributed to a higher proportion of the impact from GWP compared to UPF. The findings emphasize the importance of addressing the intake of UPF in dietary policies and recommendations to improve nutritional quality and reduce environmental impact. Furthermore, it highlights the challenges associated with classifying foods according to the NOVA classification system, particularly for staple foods in Norway such as bread.

## Supplementary Information

Supplementary Material 1.

## Data Availability

The datasets used and/or analysed during the current study are available from the corresponding author on reasonable request.
