# Supplementary material for: Nutritional quality and climate impact of Norwegian adults’ diet classified according to the NOVA system
Source: Nutr J. 2024 Dec 31;23:161. doi: 10.1186/s12937-024-01066-5 (PMC11687182; doi:10.1186/s12937-024-01066-5)
Supplement: Supplementary file 1 — Supplementary Material 1. [file 12937_2024_1066_MOESM1_ESM.docx]

**Supplementary Table S1. Additional specifications used in the present study for additives, ingredients, and preparation methods included in each NOVA group, based on the NOVA classification system [5, 7].**

|  | NOVA 1 | NOVA 2 | NOVA 3 | UPF |
| --- | --- | --- | --- | --- |
| Additives | | | | |
| Additives that may be used in food and beverages of each NOVA group | Sulphite (only in dried fruit or fruit juices)  Vitamin C in fruit juices | Enrichment with vitamins and minerals (i.e. vitamins in butter) | E200-299 (preservatives)  E300-399 (antioxidants, acidity regulators)  E500-599 (acidity regulators and anti-caking agents)  Additives between E1000-1599 that have the same properties as E200-399 and E500-599.  Enrichment with vitamins and minerals (i.e. vitamin D in milk) | E100-199 (colors)  E400-499 (emulsifiers, stabilizers, thickeners)  E600-699 (flavors and flavor enhancers)  E900-999 (glazing agents, gases, and sweeteners)  Additives between E1000-1599 that have the same properties as E100-199, E400-499, E600-699, and E900-999 |
| Ingredients | | | | |
| Added sugars | None | Sugar | Sugar labeled as sucrose, sugar-cane syrup, or sugar beet syrup on ingredient lists | Sugar in the form of inverted sugar, lactose, glucose/fructose-syrup on ingredient lists |
| Added fibers | None | None | None | All types of fibers |
| Added starches | None | Regular starch | Regular starch | Modified starch |
| Added protein isolates | None | None | None | All protein isolates added as an ingredient, including soy protein, wheat protein, wheat-gluten, and gelatin |
| Food preparation | | | | |
| Food preparation allowed in relation to food item specifications in KBS | Food codes of NOVA 1 foods boiled or baked with minimal NOVA 2 ingredients. |  | Food codes for dishes made at home which are composed of NOVA 1, 2, and 3 ingredients.  Food item codes with recipes including “exchangeable ingredients”.  Food items that do not exist as an industrially manufactured item. | Food sold from fast food outlets (for example Mc Donald’s), pre-fried foods, ready-to-heat products, and ready-to-eat products which are industrially manufactured with industrial ingredients |

Abbreviations: NOVA 1; NOVA Group 1, NOVA 2; NOVA Group 2, NOVA 3; NOVA Group 3, UPF; Ultra-Processed Foods (NOVA Group 4), KBS; food composition database and food and nutrient calculation system (Kostberegningssystemet) at the University of Oslo. [5] Monteiro, C. A. et al. Public Health Nutr. (2019). [7] Monteiro, C. et al. Public Health Nutr. (2018).

**Supplementary Table S2. Description and examples of content in each subgroup of the four NOVA groups.**

| NOVA group 1 | Unprocessed or minimally processed foods |
| --- | --- |
| Fruits and vegetables | |
| Fruits, berries, nuts and seeds | Fresh, dried, chilled and frozen fruits, nuts and seeds without salt. Includes freshly prepared smoothies, raw juice and fruit juice with only vitamin C or preservatives for longer shelf life. |
| Vegetables | Fresh, dried, chilled and frozen vegetables, root vegetables and leafy greens. Includes vegetables that have been prepared at home through boiling, baking etc. |
| Legumes | Dried, boiled, or frozen legumes, including sprouts and peas. Also includes homemade dishes of mainly legumes, like lentil soup containing mostly lentils and other NOVA 1 ingredients. |
| Potatoes | Unprocessed and minimally processed potatoes like baked, boiled or raw potatoes. |
| Bread, cake, and other grains | |
| Flour, starch, cereals, noodles, and other grain products | Unprocessed and minimally processed grains and grain products, such as quinoa, müsli, rolled oats, pasta, parboiled rice, and flour from grains. Includes grains and grain products that have been prepared at home through boiling, baking etc. |
| Fish, eggs, meat | |
| Fish and fish products | Fish and other seafood, whole, or in the form of steaks, fillets, and other cuts. Includes fish and seafood that has been prepared at home through boiling, baking, frying etc. |
| Eggs | Whole eggs in the form of boiled, fried, or otherwise prepared, as well as raw. |
| Meat and meat products | Meat, poultry, game meat, and offal, whole or in the form of cuts, steaks, fillets or minced without additives or ingredients associated to NOVA 3 or UPF. Includes meat that has been prepared at home through baking, frying, boiling, etc. |
| Dairy products | |
| Milk | Milk with different percentages of fat, pasteurized or not, without any fortification of vitamins or minerals. |
| Yoghurt and fermented milk | Plain yoghurt, soured milk, and sour cream. |
| Other dairy products | Homemade dishes consisting of mainly dairy products, and NOVA 1 ingredients. For example porridge and pancakes. Also includes cream and whipped cream. |
| Beverages | |
| Non-alcoholic beverages | Plain water, coffee, tea, coconut water. Also includes coffee with added milk. |
| Miscellaneous items | |
| Other | Herbs and spices in dried or fresh form, and plain cocoa powder. |
| NOVA group 2 | **Processed culinary ingredients** |
| Bread, cake, and other grains | |
| Flour, starch, cereals, noodles, and other grain products | Isolates of starch used in preparation of food at home, for example rice starch, potato starch and corn starch. |
| Vegetable oils and fats | |
| Vegetable oils | Oils extracted from plants (olives, corn, coconut etc). |
| Butter and margarine | Butters made from cream, or from a mix of oil and cream with additives or ingredients associated to NOVA 3. Butters could or could not contain fortification with vitamins and minerals, and could contain other NOVA 2 ingredients (i.e., salt). |
| Other fats | Lard and ghee. |
| Miscellaneous items | |
| Sugars and sweets | Sugar and sweeteners used in preparation of food, for example refined, brown, and powdered sugar, malt-extract syrup and other syrups, cocoa butter, and honey. |
| Other | Vinegar (regular and balsamic) and salt, with or without fortification of minerals (i.e iodine). |
| NOVA group 3 | **Processed foods** |
| Fruits and vegetables |  |
| Fruits, berries, nuts and seeds | Canned or bottled berries and fruits only containing additives and ingredients associated to NOVA 3, like olives and pineapple in brine. Includes fruit porridge, jams, sorbet ice cream, salted nuts, with additives and ingredients associated with NOVA 3. |
| Vegetables | Canned or bottled vegetables only containing ingredients associated to NOVA 3, like pickled vegetables, canned tomato, and vegetables preserved in oil. |
| Legumes | Beans, lentils, peas, and chickpeas in brine, tofu, and hummus, only containing additives and ingredients associated to NOVA 3. |
| Bread, cake, and other grains | |
| Bread, <25% wholegrains | Fine bread (including naan and crisps bread) made at home, at bakeries, or freshly industrial prepared bread only containing additives and ingredients associated to NOVA 3, containing less than 25% whole grain flour. |
| Bread, >25% wholegrains | Wholegrain bread (including naan and crisps bread) made at home, at bakeries, or freshly industrial prepared bread only containing additives and ingredients associated to NOVA 3, containing more than 25% whole grain flour. |
| Flour, starch, cereals, noodles, and other grain products | Industrially prepared grain products, only containing additives and ingredients associated to NOVA 3. Includes for example breakfast cereals (Weetabix, Kelloggs special K, Cornflakes), rice paper, rice noodles, egg noodles, and taco shells. Ingredient list for each product determines if the product is a NOVA 3 grain product or UPF. |
| Cakes and pastry | Freshly prepared cakes and pastry from store, bakeries or made at home, only containing additives or ingredients associated to NOVA 3. For example buns, meringue, waffles, and cakes. |
| Fish, eggs, meat | |
| Fish and fish products | Smoked, cured or salted fish and other seafood. Includes canned fish and seafood such as canned mackerel in tomato sauce and brisling (European sprat) in oil. Also includes industrially prepared dishes like sushi, fish ball and fish soup which only contain ingredients and additives associated with NOVA 3. |
| Meat and meat products | Smoked, cured or salted meat, including game meat, poultry and products made of these, only containing additives and ingredients associated to NOVA 3^1^. For example, industrially prepared meat patties, salami sausage, cured sausage, or minced meat with added salt, all without additives and ingredients associated with UPF. |
| Dairy products | |
| Milk | Milk that has been fortified with vitamin D or been lactose reduced. |
| Yoghurt and fermented milk | Biola (fermented milk) fortified with vitamin D. |
| Cheese | Fresh and fermented cheese, only containing additives and ingredients associated with NOVA 3, like cottage cheese, mozzarella, edamer “Greddost”, and Norvegia cheeses. |
| Beverages | |
| Non-alcoholic beverages | Non-alcoholic beverage only containing additives and ingredients associated to NOVA 3. For example, some plant milks, fruit and berry syrups and juices with sugar or other additives or ingredients associated to NOVA 3. |
| Alcoholic beverages | Alcoholic beverages like champagne, wine, mulled wine and beer. |
| Vegetable oils and fats | |
| Other fats | Industrially prepared sauces and fat-rich dressings containing additives and ingredients associated to NOVA 3. For example, taco sauce and pesto. |
| NOVA group 4 | **Ultra processed foods** |
| Fruits and vegetables | |
| Fruits, berries, nuts and seeds | Industrially prepared fruit and berry dishes and products that contain ingredients or additives associated with UPF. Canned and pickled fruit, berries, and olives, including products like compote, jam, and jelly, containing additives and ingredients associated to UPF. |
| Vegetables | Canned and pickled vegetables, instant-soups, pre-packed vegetable products (for example, sauerkraut, vegetable patties, spreads from vegetables), all containing additives or ingredients associated with UPF. |
| Legumes | Industrially prepared tomato beans, burgers, hot dogs, minced dough, and yoghurt made of soybeans, spread made of chickpeas, all containing additives and ingredients associated to UPF. |
| Potatoes | Industrially prepared potato dishes like, store bought potato salad, mashed potato from powder, pommes frites from stores or fast-food restaurants/outlets, gnocchi, or semi-finished potato gratin, which contains additives and ingredients associated to UPF. |
| Bread, cake, and other grains | |
| Bread, <25% wholegrains | Industrially prepared fine bread (including naan and crisps bread) containing additives and ingredients associated to UPF, containing less than 25% whole grain flour. |
| Bread, >25% wholegrains | Industrially prepared dark bread (including naan and crisps bread) containing additives and ingredients associated to UPF, containing more than 25% whole grain flour. |
| Flour, starch, cereals, noodles, and other grain products | Breakfast cereals, granola, muesli, pasta, instant noodles, industrially prepared tortillas, savory cookies, and porridges containing additives and ingredients associated to UPF. |
| Cakes and pastry | Industrially prepared cakes, pastry, sweet cookies, and other sweet buns, like croissant, gingerbread, donuts, waffles, macaroons, and muffins, all containing additives or ingredients associated to UPF. |
| Fish, eggs, meat | |
| Fish and fish products | Industrially prepared products of fish and other seafood, containing additives or ingredients associated to UPF, for example pickled herring in mustard, or canned mackerel in tomato sauce. Also includes pre-heated or ready-to-heat products like fried shrimps, fish “cakes”, “sticks”, and “pudding”, fish gratin, caviar, and instant fish soup. |
| Meat and meat products | Industrially prepared products of meat, (including poultry, game meat, and offal), containing additives or ingredients with UPF. Includes ready-to-eat or ready-to-heat products and dishes, for example sausages (used for dinner and for spreads), meatballs, bacon, liver or lung paste, meat cuts, lasagna, and meat stews. |
| Dairy products | |
| Milk | Milk or milk-based products containing additives and ingredients associated to UPF, for example, chocolate milk, and milk-based hot chocolate drink. |
| Yoghurt and fermented milk | Industrially prepared yoghurt, yoghurt-based products, and fermented milk containing colors and artificial sweeteners, or other additives and ingredients associated with UPF (substances of no or rare culinary use, such as hydrogenated oils, protein isolates like gelatin and soy protein, glucose fructose syrup and fiber isolates [5, 7]), for example vanilla yoghurt, biola with raspberry, and industrially manufactured tzatziki. |
| Cheese | Cheese and products of cheese that contain additives and ingredients associated to UPF, for example quark (“kesam”) with vanilla flavor, soft spread cheeses with flavor of shrimp, bacon, or tomato, Philadelphia cheese, cream cheeses and “Primost” (a Norwegian cream whey cheese). |
| Other dairy products | Other dairy dishes and products of dairy, like cream sauces, cheddar sauce, vanilla sauce, chocolate pudding, cream on box, and dairy ice cream, all containing additives or ingredients associated to UPF. |
| Beverages | |
| Non-alcoholic beverages | Non-alcoholic beverages, containing additives or ingredients associated with UPF, for example soda, carbonated water, some types of plant milk, fruit and berry syrups containing artificial sweeteners, alcohol-free beer, flavoured tea, and coffee mocha. |
| Alcoholic beverages | Alcoholic beverages like liquor and carbonated cider with flavor. |
| Vegetable oils and fats | |
| Butter and margarine | Butters and margarines containing additives and ingredients associated to UPF, for example margarines like Olivero, Brelett and Vita. |
| Other fats | Industrially prepared mayonnaise-based spreads, like shrimp salad, Italian salad, or beetroot salad, aioli, fatty dressings like thousand island, and remoulade, all containing additives and ingredients associated to UPF. |
| Miscellaneous items | |
| Sugars and sweets | Industrially manufactured sweet snacks and “candy” containing additives or ingredients associated to UPF, such as chocolate bars, chocolate spreads and chocolate sauce, jelly, sauces for dessert, low-calorie/calorie-free sugars and syrups, wine gum, marshmallows, gum, and lozenges. |
| Salty snacks | Industrially manufactured savoury snacks of grains, potato, or nuts, containing additives or ingredient associated to UPF or which is pre-fried. For example, tortilla chips of corn, potato chips, chili nuts and popcorn. |
| Other | Industrially prepared sauces, dressings and miscellaneous products used in preparation of food and meals, which contain additives or ingredients associated to UPF. In example mustard, tomato ketchup, cheddar sauce, gravy, barbeque sauce, pesto, or wok sauce. Also includes miscellaneous products like protein bars and meal replacement shakes like Herbalife, containing additives and ingredients associated to UPF. |

Ingredients associated to NOVA 3 are substances from NOVA 1 and 2. NOVA 3 additives are substances added to prolong product duration, protect original properties, or prevent the proliferation of microorganisms (E200-299 (preservatives), E300-399 (antioxidants, acidity regulators), E500-599 (acidity regulators and anti-caking agents), and additives between E1000-1599 that have the same properties as E200-399 and E500-599 [27]. UPF ingredients are substances of no or rare culinary use, such as hydrogenated oils, protein isolates like gelatin and soy protein, glucosefructose syrup and fiber isolates [5, 7]. UPF additives are additives E100-199 (colors), E400-499 (emulsifiers, stabilizers, thickeners), E600- 699 (flavors and flavor enhancers), E900-999 (glazing agents, gases and sweeteners), and additives between E1000-1599 that have the same properties as E100-199, E400-499, E600-699 and E900-999. Processes associated to UPF is pre-frying and formation of ready-to-heat products [5, 7]. Abbreviations; UPF, Ultra-processed foods (NOVA Group 4 foods). [5] Monteiro, C. A. et al. Public Health Nutr. (2019). [7] Monteiro, C. et al. Public Health Nutr. (2018).

**Supplementary Table S3.** The absolute intake in grams from foods classified according to the four NOVA groups among Norwegian adults (n=348)

| **Food group** | **Intake in grams**  Median (P25, P75)  mean | | | |
| --- | --- | --- | --- | --- |
|  | **NOVA 1** | **NOVA 2** | **NOVA 3** | **UPF** |
| **Total intake** | 3254 (2680, 3984)  3428 | 6 (0.2, 18)  13 | 355 (173, 588)  437 | 789 (518, 1111)  906 |
| **Fruits, vegetables**, **and potatoes** | 830 (422, 1338)  991 | - | 0 (0, 32)  30 | 15 (0, 54)  47 |
| Fruits and berries | 155 (50, 303)  538 | - | 0 (0, 8)  7 | 0 (0, 20)  21 |
| Vegetables and legumes | 100 (50, 169)  126 | - | 0 (0, 12)  22 | 0 (0, 0)  14 |
| Potatoes | 0 (0, 85)  44 | - | - | 0 (0, 0)  12 |
| **Bread, cake, and other grain products** | 0 (0, 75)  50 | 0 (0, 0)  0.07 | 24 (0, 77)  50 | 93 (45, 173)  117 |
| Bread | - | - | 10 (0, 48)  33 | 79 (25, 143)  94 |
| Flour, cereals, noodles,     and other grain products | 0 (0, 75)  50 | 0 (0, 0)  0.07 | 0 (0, 0)  6 | 0 (0, 30)  23 |
| Cakes and pastry | - | - | 0 (0, 0)  11 | 0 (0, 40)  26 |
| **Fish, eggs, and meat** | 56 (0, 123)  78 | - | 0 (0, 37)  34 | 53 (17, 126)  96 |
| Fish and fish products | 0 (0, 0)  20 | - | 0 (0, 8)  23 | 0 (0, 17)  29 |
| Eggs | 0 (0, 28)  21 | - | - | - |
| Meat and meat products | 0 (0, 69)  37 | - | 0 (0, 7)  12 | 28 (0, 17)  29 |
| **Dairy products** | 18 (0, 170)  111 | - | 40 (14, 110)  105 | 3 (0, 63)  49 |
| Milk, yoghurt, and creams | 18 (0, 170)  111 | - | 0 (0, 25)  66 | 0 (0, 61)  36 |
| Cheese | - | - | 26 (10, 50)  39 | 0 (0, 0)  4 |
| Ice cream and dessert | - | - | - | 0 (0, 0)  8 |
| **Beverages** | 1270 (803, 1918)  1394 | - | 0 (0, 160)  118 | 323 (121, 625)  451 |
| Non-alcoholic beverages | 1270 (803, 1918)  1394 | - | 0 (0, 0)  26 | 320 (120, 623)  447 |
| Alcoholic beverages | - | - | 0 (0, 80)  92 | 0 (0, 0)  4 |
| **Vegetable oils and fats** | - | 3 (0, 11)  8 | 0 (0, 0)  4 | 21 (8, 47)  31 |
| Butter, margarine, vegetable oil | - | 3 (0, 11)  8 | - | 8 (0, 19)  14 |
| Other fats | - | - | 0 (0, 0)  4 | 4 (0, 28)  17 |
| **Miscellaneous items** | 0 (0, 4)  1 | 0 (0, 5)  4 | - | 16 (2, 45)  35 |
| Sugars and sweets | - | 0 (0, 4)  4 | - | 11 (1, 33)  24 |
| Salty snacks | - | - | - | 0 (0, 6)  10 |
| Other | 0 (0, 4)  1 | 0 (0, 0.3)  0.7 | - | 0 (0, 0)  1 |
| **Composite dishes** | - | - | 32 (0, 150)  97 | 0 (0, 70)  50 |
| Homemade dishes | - | - | 32 (0, 150)  97 | - |
| Pizza, kebab, hamburgers | - | - | - | 0 (0, 70)  50 |

Full disclosure of food items included in each food group can be found in Supplementary Table S1.  
Abbreviations: NOVA 1; NOVA Group 1, NOVA 2; NOVA Group 2, NOVA 3; NOVA Group 3, P25; 25^th^ percentile, P75; 75^th^ percentile, UPF; Ultra-processed foods (NOVA Group 4). 

**Supplementary Table S4. Nutrient density in total diet and according to NOVA group for other nutrients not included in the main table.**

| Nutrient | Nutrient density  Median (P25, P75)  Mean | Nutrient density according to NOVA group  Median (P25, P75)  Mean | | | | | |  |
| --- | --- | --- | --- | --- | --- | --- | --- | --- |
|  |  | **Main analysis** | | | | **Sensitivity analysis** | | |
|  |  | **NOVA 1** | **NOVA 2** | **NOVA 3** | **UPF** | **NOVA 3** | **UPF** | |
| Omega-3,  E% | 1.0 (0.7, 1.5)  1.2 | 0.7 (0.4, 1.1)  1.1 | 0.4 (0.0, 2.7)  1.3 | 0.4 (0.2, 1.0)  1.0 | 1.0 (0.7, 1.6)  1.3 | 0.5 (0.3, 1.0)  0.9 | 1.1 (0.7, 1.8)  1.4 | |
| Omega-6,  E% | 4.7 (3.8, 5.6)  4.9 | 4.1 (2.6, 5.6)  4.4 | 0.7 (0.0, 8.2)  5.3 | 2.4 (1.1, 4.3)  3.5 | 5.2 (4.0, 6.6)  5.4 | 2.5 (1.5, 4.2)  3.5 | 5.4 (4.1, 7.0)  5.7 | |
| Thiamine (B1),  mg/MJ | 0.2 (0.1, 0.2)  0.2 | 0.2 (0.2, 0.3)  0.3 | 0.0 (0.0, 0.0)  0.0 | 0.1 (0.1, 0.2)  0.2 | 0.2 (0.1, 0.2)  0.2 | 0.2 (0.1, 0.2)  0.2 | 0.1 (0.1, 0.2)  0.2 | |
| Riboflavin (B2),  mg/MJ | 0.2 (0.2, 0.3)  0.2 | 0.4 (0.3, 0.5)  0.5 | 0.0 (0.0, 0.0)  0.0 | 0.2 (0.1, 0.3)  0.2 | 0.1 (0.1, 0.2)  0.2 | 0.2 (0.1, 0.2)  0.2 | 0.1 (0.1, 0.2)  0.2 | |
| Niacin (B3),  mg/MJ | 2.1 (1.7, 2.8)  2.4 | 3.2 (2.3, 5.0)  3.9 | 0.0 (0.0, 0.0)  0.0 | 1.3 (0.7, 2.2)  1.7 | 1.6 (1.2, 2.2)  1.9 | 1.5 (0.9, 2.1)  1.8 | 1.5 (1.1, 2.2)  1.9 | |
| Pyridoxine (B6),  mg/MJ | 0.2 (0.1, 0.2)  0.2 | 0.3 (0.2, 0.4)  0.4 | 0.0 (0.0, 0.0)  0.0 | 0.1 (0.1, 0.2)  0.1 | 0.1 (0.1, 0.1)  0.1 | 0.1 (0.1, 0.2)  0.1 | 0.1 (0.1, 0.2)  0.1 | |
| Potassium (K),  g/MJ | 0.4 (0.4, 0.5)  0.4 | 0.9 (0.7, 1.1)  1.0 | 0.0 (0.0, 0.0)  0.0 | 0.3 (0.2, 0.4)  0.3 | 0.3 (0.2, 0.3)  0.3 | 0.3 (0.2,0.4)  0.3 | 0.3 (0.2, 0.3)  0.3 | |
| Magnesium,  mg/MJ | 39 (33, 45)  40 | 56 (44, 68)  58 | 0.0 (0.0, 0.0)  1.1 | 39 (31, 50)  42 | 31 (26, 37)  33 | 41 (34, 50)  43 | 29 (24, 36)  32 | |
| Zink,  mg/MJ | 1.2 (1.0, 1.5)  1.3 | 1.3 (1.0, 1.6)  1.4 | 0.0 (0.0, 0.0)  0.0 | 1.7 (1.2, 2.2)  1.7 | 1.0 (0.8, 1.3)  1.1 | 1.6 (1.2, 2.0)  1.6 | 1.0 (0.8, 1.3)  1.1 | |
| Copper,  mg/MJ | 0.1 (0.1, 0.2)  0.1 | 0.2 (0.1, 0.2)  0.2 | 0.0 (0.0, 0.0)  0.0 | 0.1 (0.1, 0.2)  0.1 | 0.1 (0.1, 0.2)  0.1 | 0.1 (0.1, 0.2)  0.1 | 0.1 (0.1, 0.1)  0.1 | |
| Phosphorus,  g/MJ | 0.2 (0.2, 0.2)  0.2 | 0.3 (0.2, 0.3)  0.3 | 0.0 (0.0, 0.0)  0.0 | 0.3 (0.2, 0.3)  0.3 | 0.2 (0.1, 0.2)  0.2 | 0.2 (0.2, 0.3)  0.2 | 0.1 (0.1, 0.2)  0.2 | |
| Manganese,  mg/MJ | 0.0 (0.0, 0.0)  0.0 | 0.0 (0.0, 0.0)  0.0 | 0.0 (0.0, 0.0)  0.0 | 0.0 (0.0, 0.0)  0.0 | 0.0 (0.0, 0.0)  0.0 | 0.0 (0.0, 0.0)  0.0 | 0.0 (0.0, 0.0)  0.0 | |

Nutrient density is measured as percentage of energy intake, or as gram/mg/µg pr Mega Joule (MJ). Abbreviations: NOVA 1; NOVA Group 1, NOVA 2; NOVA Group 2, NOVA 3; NOVA Group 3, P25; 25^th^ percentile, P75; 75^th^ percentile, UPF; Ultra-Processed Foods (NOVA Group 4).
